# Curcumin modulation of L-dopa and rasagiline-induced neuroprotection in rotenone model of Parkinson’s disease

**DOI:** 10.22038/IJBMS.2022.61687.13650

**Published:** 2023-02

**Authors:** Marwa El-Sayed El-Shamarka, Omar ME Abdel-Salam, Nermeen Shafee, Hala M Zeidan

**Affiliations:** 1 Department of Narcotics, Ergogenics, and Poisons, Medical Research Division, National Research Centre, Cairo, Egypt; 2 Department of Pathology, Medical Research Division, National Research Centre, Cairo, Egypt; 3 Department of Research on Children with Special Needs, Medical Research Division, National Research Centre, Cairo, Egypt

**Keywords:** Curcumin, L-dopa, Neurodegeneration, Oxidative stress, Parkinson’s disease, Rasagiline, Rotenone

## Abstract

**Objective(s):**

Parkinson’s disease (PD) is one of the most incurable, chronic, and progressive neurodegenerative disorders Worldwide. Curcumin, a natural polyphenolic anti-oxidant compound, has a long history in traditional medicine. We investigate the effect of curcumin on brain oxidative stress, DNA fragmentation, and motor changes in rotenone-induced PD in mice. The possible modulation of the anti-parkinsonian action of drugs L-dopa and rasagiline by curcumin was also studied.

**Materials and Methods:**

Mice received rotenone 1.5 mg/kg and were treated with curcumin (150 mg/kg), L-dopa (25 mg/kg), rasagiline (1 mg/kg), L-dopa+curcumin, or rasagiline+curcumin. Striatal malondialdehyde, reduced glutathione, nitric oxide, tyrosine hydroxylase, and brain DNA fragmentations were measured. Histopathological examination of brain tissues was done. Motor coordination and behavioral tests such as wire-hanging, stair, and wood-waking tests were included.

**Results:**

Rotenone caused elevation in brain malondialdehyde and nitric oxide contents, depletion of reduced glutathione accompanied by a reduction in rearing behavior, and impairment of motor activity in wire-hanging, stair, and wood-waking tests. Also, severe DNA fragmentation in the striatum, marked decrease of substantia nigra pigmented neurons, neuronal degeneration in the cerebral cortex and hippocampus, decreased glial fibrillary acidic protein reaction (GFAP) and glial cell size in the cerebral cortex were caused by rotenone. In rotenone-treated mice, brain oxidative stress was decreased by curcumin, L-dopa, rasagiline, curcumin+L-dopa, and curcumin+rasagiline. These treatments also prevented DNA fragmentation and markedly improved the motor and behavioral impairment caused by rotenone. Rotenone-induced histopathological changes were ameliorated by curcumin which had an additive effect to that of l-dopa or rasagiline.

**Conclusion:**

These data indicate that curcumin showed additive neuroprotective effects to L-dopa or rasagiline and ameliorated neurodegeneration, DNA fragmentation, and motor defects caused by rotenone in mice.

## Introduction

Parkinson’s disease (PD) is one of the most incurable, chronic, and progressive neurodegenerative disorders worldwide. It is the second neurological geriatric disease after Alzheimer’s disease and affects about 6.3 million people over the age of 65 (1). PD is characterized by two main neuropathological defects; dopaminergic neuron degeneration or loss in the substantia nigra and the existence of Lewy bodies with fibrous, insoluble aggregates of α-synuclein in the surviving central and peripheral nervous system neurons (2). It is associated with motor dysfunction as resting tremors, rigidity, and postural instability (3). In PD non-motor manifestations also occur including autonomic disturbances and neuropsychiatric symptoms such as depression which may account for morbidity. Pathogenetic processes such as neuroinflammation and oxidative stress are most likely the cause of dopaminergic cell death in PD.

L-3,4-dihydroxyphenylalanine (L-DOPA) is an efficient therapy in PD treatment as it increases dopamine content in the brain (4). It is the immediate precursor of dopamine and is used to correct the deficit in striatal dopamine that results from the death of the substantia nigra dopaminergic neurons; the main neuropathologic event in PD. L-dopa is transported to the brain and decarboxylated to dopamine. It is given in the form of “Sinemet” together with carbidopa, a decarboxylase inhibitor, to prevent its peripheral decarboxylation (5). The main goal for the future cure of PD is the discovery and development of neuroprotective medications to terminate the disease progression and reduce the toxicity of L-DOPA. 

Selegiline, a monoamine oxidase (MAO) type B inhibitor that stops dopamine metabolism in the brain, is used as a medication in the treatment of PD and major depressive disorder. Rasagiline (N-propargyl-1 (R)-aminoindan) is a highly selective, irreversible monoamine oxidase type B (MAO-B) inhibitor that is used as an antiparkinson drug either alone (monotherapy) in early disease or in combination with L-DOPA (adjuvant therapy) in the more advanced stages. Rasagiline is preferable to selegiline because it is not metabolized to amphetamine compounds. It relieves the motor symptoms of PD and increases dopamine levels by inhibiting the nigrostriatal dopamine metabolism irreversibly, thus giving symptomatic benefits (6).  

Curcumin (diferuloylmethane), is a natural polyphenolic anti-oxidant compound extracted from the root of the perennial herb *Curcuma longa*, known as turmeric, a yellow spice that has a long history in traditional medicine (7). It has also been suggested that curcumin may reduce the incidence of PD, as an epidemiological study has shown that Indian populations that widely consume curcumin lack age-related changes in dopaminergic neurons of their substantia nigra (8). Curcumin crosses the blood-brain barrier and has anti-oxidant and anti-inflammatory potential for treating neurodegenerative diseases such as Parkinson’s and Alzheimer’s diseases (9).  Also, curcumin ameliorates neurobehavioral disturbances and brain oxidative stress in boldenone, an anabolic androgenic steroid used in veterinary medicine, treated rats (10). Natural anti-oxidants such as curcumin could be helpful in the management of PD and in reducing the toxicity of L-DOPA. 

Different animal models, including the rotenone model, have been used to investigate the underlying mechanisms of PD pathogenesis and try new neuroprotective medications (11). Rotenone is a highly lipophilic insecticide that can cross freely the blood-brain barrier, without depending on a specific transporter, and cause neurotoxicity in experimental animals (12). Betarbet *et al*. (2000) found that systemic rotenone could reproduce the two neuropathological hallmarks together with the motor deficits of PD (13). Uversky (2004) reported that rotenone neuronal injury occurred via various pathophysiological mechanisms such as oxidative damage due to production of ROS, microglial activation, α-synuclein aggregation in different brain regions, and apoptosis (14).

The present study aimed to investigate the effect of curcumin on brain oxidative stress, DNA fragmentation, and motor changes in rotenone-induced PD in mice. The possible modulation of the action of the anti-parkinsonian drugs L-dopa and rasagiline by curcumin was also studied.

## Materials and Methods

***Animals***

Male Swiss albino mice (20-25 g: National Research Centre, Cairo) were used in the experiments. Mice were housed under standard conditions of temperature and humidity with a 12-hr light/dark cycle and had free access to food and water. The protocols used in this study complied with The Guide for Care and Use of Laboratory Animals published by the US National Institutes of Health (NIH Publication No. 85-23, revised 2011) and were approved by the National Research Centre Ethical Committee for Medical Research (Animal Experimentation Sector). All efforts were made to minimize animal suffering and reduce the number of animals used.

***Chemicals and reagents***

Rotenone (lot no: 021M2227V) was obtained from Sigma (St Louis, MO, USA) and dissolved in dimethyl sulfoxide (DMSO). Rotenone was given subcutaneously (SC) at a dose of 1.5 mg/kg, three times per week for 2 weeks. Curcumin (lot no: SHBL0796) was obtained from Sigma (St Louis, MO, USA) and dissolved in olive oil, and given at an oral dose of 150 mg/kg/day for 14 days. Levodopa/Carbidopa (Sinemet 25/250) was dissolved in saline and given at a dose of 25 mg/kg/day for 14 days. Rasagiline (Parkintreat, 1 mg) was dissolved in saline and given at a dose of 1 mg/kg/day for 14 days.

***Experimental design***

Seven groups of male albino mice were used. The control group (DMSO) received DMSO SC and olive oil orally. The other six groups were treated with rotenone 1.5 mg/kg, SC, every other day for two weeks. Starting from the first day of rotenone injection, 5 groups of rotenone were also treated with either curcumin (150 mg/kg), L-dopa (25 mg/kg), rasagiline (1 mg/kg), L-dopa+curcumin, or rasagiline+curcumin.

By the end of the experiment, all animals were sacrificed using cervical dislocation. Animals were quickly dissected, brains were rapidly excised on ice, and striatum regions were isolated and kept in sterile tubes at -80 °C until assayed. 20 mg of brain striatum tissue was cut into small pieces and then collected into a 1.5 ml sterile microcentrifuge tube for DNA extraction.

***Neurochemical analyses***

*Determination of lipid peroxidation*

Malondialdehyde (MDA), a measure of lipid peroxidation, was estimated according to the method of Ruiz-Larrea *et al*. (1994) (15). The absorbance of the resultant pink product was measured at 532 nm in the striatal tissue homogenate. 

*Determination of reduced glutathione*

Ellman’s method was used to measure GSH level in the striatal tissue homogenate. The absorbance of the intense yellow color was measured spectrophotometrically at 412 nm (16).

*Determination of nitric oxide*

Nitric oxide was determined colorimetrically in the striatal tissue homogenate according to the method described by Moshage *et al*. (1995) (17). The absorbance of the resultant deep purple azo compound was read at 540 nm.

*Determination of striatal tyrosine hydroxylase enzyme*

The levels of Tyrosine hydroxylase enzyme in the striatal homogenate were measured using ELISA kits (#:96791) from the Glory Science Co., (China).

***Behavioral tests***

*Cylinder test*

The cylinder test was used to assess spontaneous forelimb use. Mice are placed in a transparent Plexiglas cylinder and the number of spontaneous rears made during 5 min in the cylinder was measured for each animal (18).

*Wire-hanging test*

To evaluate motor strength, mice were made to hang by their forelimbs from a steel rod (25 cm long, 0.2 cm in diameter) placed 0.25 m above the bench. The time each mouse could hang suspended from the rod was recorded for three trials with a cut-off time of 180 sec (19).

*Wood-walking test*

To assess motor coordination, mice were made to walk over a wooden stick (~0.5 m in length, 1 cm in width), and the time each mouse spent to reach the end is recorded (20).

*Stair test*

In order to assess skilled reaching, mice were placed at the bottom of a stair (30 cm in length) placed at an angle of 55° above the bench, and the latency to climb the stair was recorded for each mouse (21).

***DNA ladder assay***

Genomic DNA was extracted from mice’s brain striatum by using a ready-made kit (Gene Jet Genomic DNA Purification kit, Thermo Scientific, Lithuania) according to the manufacturer’s instructions. Extracted DNA was quantified by using a nanodrop spectrophotometer device (2000 C, Thermo Scientific, USA). Five micrograms of DNA samples were subjected to electrophoresis in 1.5% agarose gel containing 1.0 μg/ml ethidium bromide. DNA was then visualized and photographed in agarose gel by using a gel documentation system (22, 23).

***Histopathological studies***

Brain samples were fixed in 10 % neutral-buffered formalin saline for 72 hr at least. All the specimens were washed in tap water for half an hour and then dehydrated in ascending grades of alcohol, cleared in xylene, and embedded in paraffin. Serial sections 6 μm thick were cut and stained with hematoxylin and eosin for histopathological investigation.

***Immunohistochemistry for glial fibrillary acidic protein***

Paraffin-embedded brain sections were deparaffinized and hydrated. Immunohistochemistry was performed with a mouse monoclonal glial fibrillary acidic protein (GFAP) for detection of the GFAP activity. The paraffin sections were heated in a microwave oven (25 min at 720 W) for antigen retrieval and incubated with anti-GFAP antibodies (1:50 dilution) overnight at 4 ^°^C. After washing with PBS, followed by incubation with biotinylated goat-anti-rabbit- immunoglobulin G secondary antibodies (1:200 dilution; Dako Corp.) and streptavidin/alkaline phosphatase complex (1:200 dilution; Dako) for 30 min at room temperature, the binding sites of antibody were visualized with DAB (Sigma). After washing with PBS, the samples were counterstained with H&E for 2-3 min and dehydrated by transferring them through increasing ethanol solutions (30%, 50%, 70%, 80%, 95%, and 100% ethanol). Following dehydration, the slices were soaked twice in xylene at room temperature for 5 min, mounted, examined, and evaluated by a high-power light microscope. Images were examined and photographed under a digital camera (Microscope Digital Camera DP70, Tokyo, Japan), and processed using Adobe Photoshop version 8.0.

***Statistical analysis***

Data were subjected to statistical analysis using Statistical Package for Social Science (SPSS 2016). Data are represented as the mean±SEM. Simple one-way analysis of variance was performed to identify the effect of treatment on the studied parameters. Duncan’s multiple-range test was performed to distinguish between different and significant means at *a P*-value less than 0.05.

## Results

***Biochemical results***

*Lipid peroxidation*

**Figure 1 (A-C):**
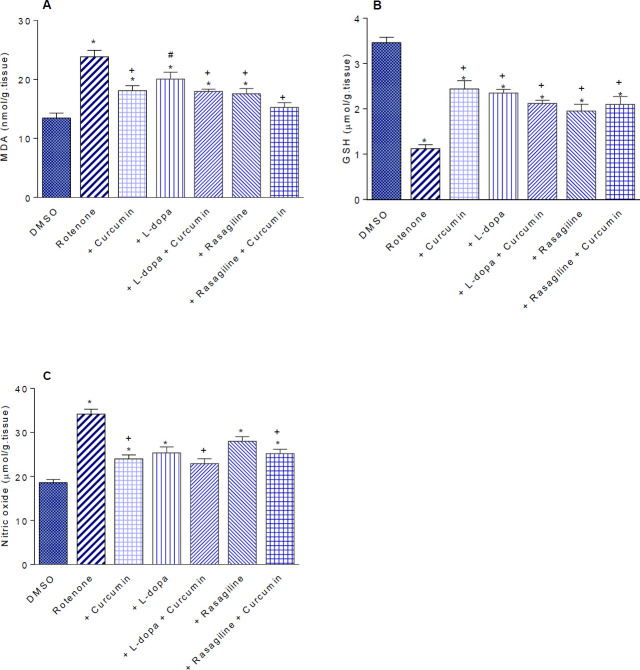
Effect of curcumin on (A) malondialdehyde, (B) reduced glutathione, and (C) nitric oxide in the brain of mice treated with rotenone alone or rotenone combined with L-dopa or rasagiline

Rotenone significantly increased brain malondialdehyde (MDA) by 77% compared with the vehicle control value (23.84±1.1 vs 13.47±0.83 nmol/g.tissue). Administration of curcumin resulted in a 24.1% decrease in brain MDA compared with the rotenone-only group (18.1±0.84 vs 23.84±1.1 nmol/g.tissue). Mice given either L-dopa or rasagiline showed 15.7% and 26.2% decrements in brain MDA, while co-administering curcumin with either L-dopa or rasagiline caused 24.7% and 36.2% decreases in MDA compared with the rotenone only group (Figure 1A). 

*Reduced glutathione*

Rotenone-treated mice had significantly decreased brain GSH concentrations by 67.6% as compared with the vehicle group (1.12±0.09 vs 3.46±0.12 µmol/g.tissue). Curcumin given to rotenone-treated mice caused a significant increase in brain GSH by 117.8% (2.44±0.18 vs 1.12±0.09 µmol/g.tissue). Administration of L-dopa or rasagiline alone or in combination with curcumin did not result in increased brain GSH compared with that caused by curcumin (Figure 1B).

*Nitric oxide*

Rotenone caused an 83.9% increase in brain nitric oxide level as compared with the vehicle control group (34.2±1.1 vs 18.6±0.71 µmol/g.tissue). A 29.8% decrease in brain nitric oxide was observed after curcumin administration compared with the rotenone control group (24.0±0.94 vs 34.2±1.1 µmol/g.tissue). Administration of L-dopa or rasagiline alone or in combination with curcumin did not result in a significant change in brain nitric oxide compared with the curcumin-only treated group (Figure 1C).

*Striatal tyrosine hydroxylase*

**Figure 2:**
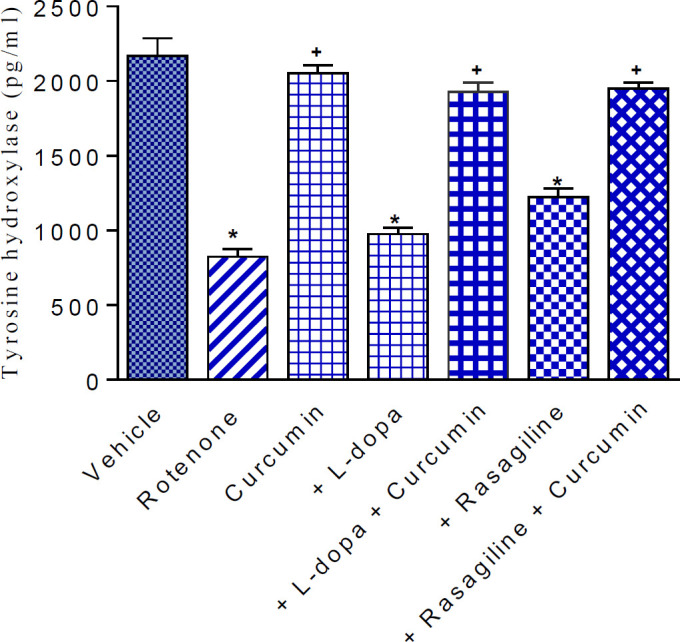
Effect of curcumin, L-dopa, and rasagiline on striatal tyrosine hydroxylase content in mice treated with rotenone

Compared with the vehicle group, injection of rotenone caused significant depletion of tyrosine hydroxylase in the striatum by 61.9% (824.8±48.4 vs 2167±119 pg/ml). Curcumin given to rotenone-treated rats resulted in a marked increase in tyrosine hydroxylase content by 149% compared with the rotenone-only group (2054.1±52.6 vs 824.8±48.4 pg/ml). Striatal tyrosine hydroxylase showed 18.4% and 48.1% increments after treatment with either L-dopa or rasagiline, respectively (976.4±41.3 and 1221.4±60.0 vs 824.8±48.4 pg/ml). Mice co-administered curcumin with either L-dopa or rasagiline showed increases in tyrosine hydroxylase by 133.4% and 136.6% compared with the rotenone-only group (Figure 2). 

***Behavioral results***

*Cylinder test*

**Figure 3 (A-D):**
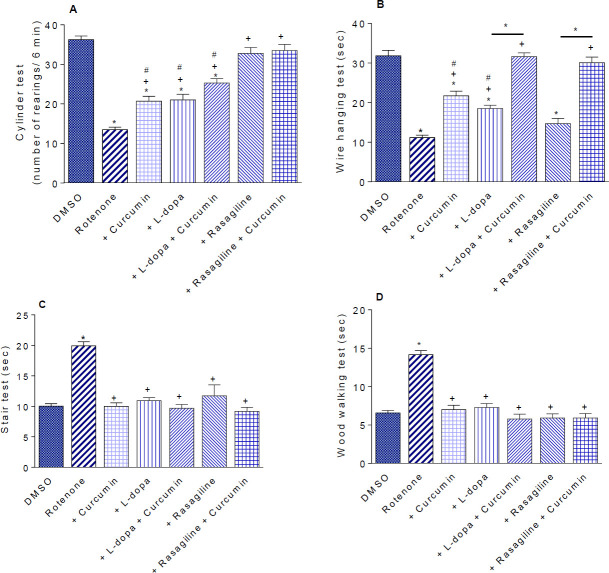
Effect of different treatments on rearing activity (cylinder test), latency to hang suspended from a steel rod (wire hanging test), latency to ascend a stair (stair test), latency to traverse a wooden stick (wood walking test)

Rotenone decreased rearing behavior by 62.7% (13.5±0.63 vs 36.25±0.94; rotenone vs vehicle). In rotenone-treated mice, rearing increased by 53.3% by curcumin (20.7±1.22 vs 13.5±0.63). Rearing increased by 55.5% and 87.4% by L-dopa and rasagiline, respectively. The decrease in rearing caused by rotenone was almost reversed after administration of curcumin with either L-dopa or rasagiline (Figure 3A).

*Wire-hanging test*

Mice given rotenone exhibited significantly less time to hang from a wire compared with their vehicle-treated counterparts (64.9% decrease in time; 11.17±0.63 vs 31.83±1.4 sec). Administration of curcumin or L-dopa resulted in 94.3% and 65.8% increases in the time spent by the mouse compared with the rotenone control group (21.7±1.19 and 18.52±0.84 vs 11.17±0.63 sec). The rotenone-induced grip strength deficit was almost reversed after administration of curcumin with either L-dopa or rasagiline (Figure 3B).

*Stair test*

The time spent by the mouse to ascend the stairs was significantly increased by 99.3% after rotenone injection compared with the vehicle group (19.93±0.68 vs 10.0±0.41 sec). Administration of curcumin, L-dopa, rasagiline, or their combinations resulted in reversing the effect of rotenone (Figure 3C).

*Wood-walking test*

The time the mouse took to reach the end of the wooden stick increased by 115.3% from 6.58±0.31 sec in the vehicle group to 14.17±0.52 sec in the rotenone-only group. No significant change in time spent by mice in reaching the end of the wooden stick was observed after treatment with curcumin, L-dopa, rasagiline, or their combinations as compared with the rotenone control group (Figure 3D).

***DNA ladder assay***

**Figure 4:**
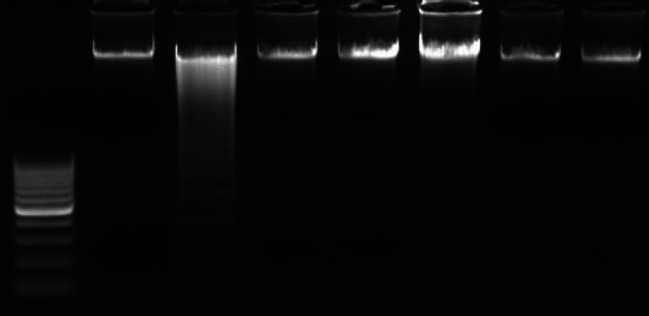
DNA fragmentation was assessed in mice brain striatum of all experimental groups. Lane 1: DNA ladder, lane 2: control group, lane3: rotenone-treated group, lane 4: curcumin with the rotenone-treated group, lane 5: L-dopa with the rotenone-treated group, lane 6: Rasagiline with the rotenone-treated group, lane 7: L-dopa, curcumin, and rotenone-treated group, lane 8: Rasagiline, curcumin, and rotenone-treated group

Figure (4) illustrates the effects of curcumin, rasagiline, and L-3,4-dihydroxyphenylalanine (L-dopa) and their combinations on DNA fragmentation in brain striatum tissues of rotenone-treated mice. DNA ladder assay shows DNA fragmentation in ladder pattern upon rotenone treatment. However, DNA damage was quenched in the present study by co-treatments with curcumin, rasagiline, and L-dopa in rotenone-treated mice (lanes 4, 5, and 6, respectively). Additionally, co-treatments by curcumin in combination either with rasagiline or L-dopa in rotenone-treated mice (lanes 7 and 8, respectively) showed a protective effect on DNA that appeared as a good intact band without fragmentation. 

***Histopathological results***

**Figure 5:**
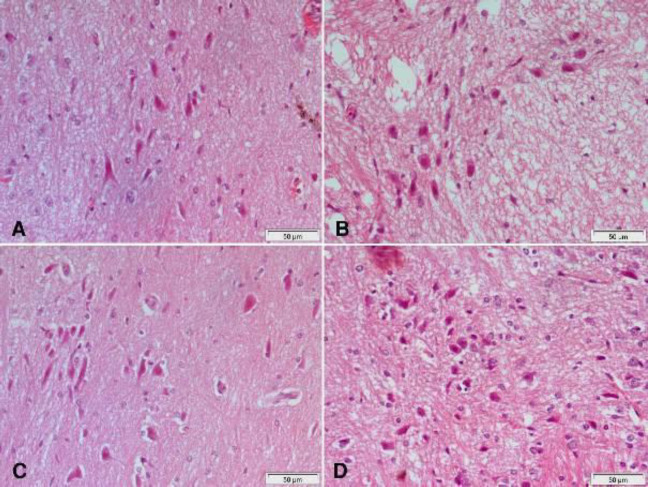
Representative photomicrographs of substantia nigra area sections stained with Hx & E

**Figure 6:**
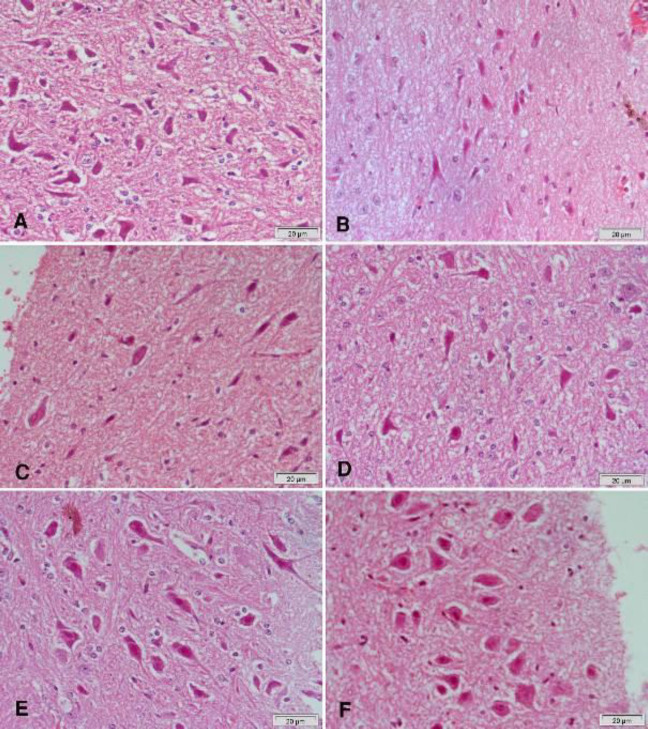
Representative photomicrographs of substantia nigra area sections stained with Hx & E

**Figure 7:**
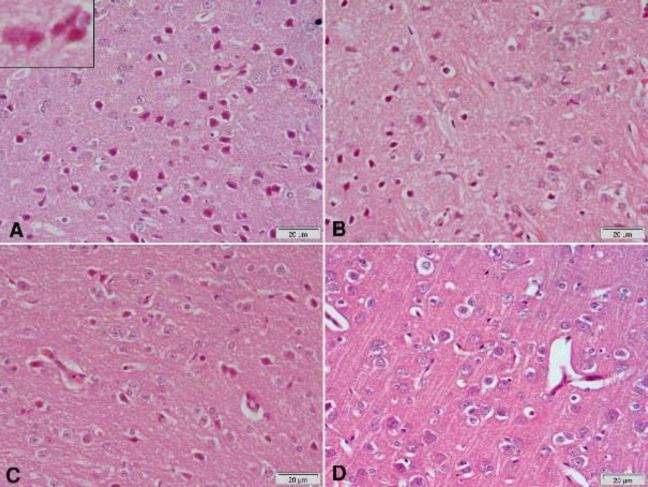
Representative photomicrographs of cerebral cortex sections stained with Hx & E

**Figure 8:**
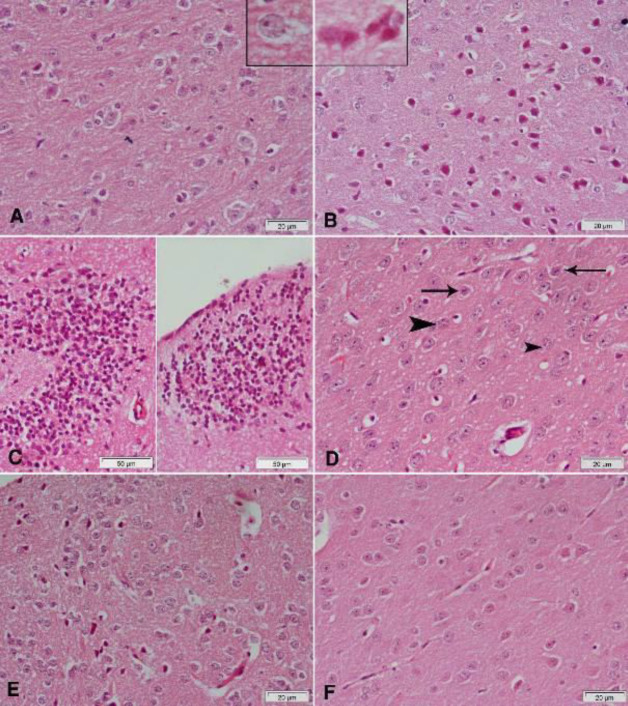
Representative photomicrographs of cerebral cortex sections stained with Hx & E

**Figure 9:**
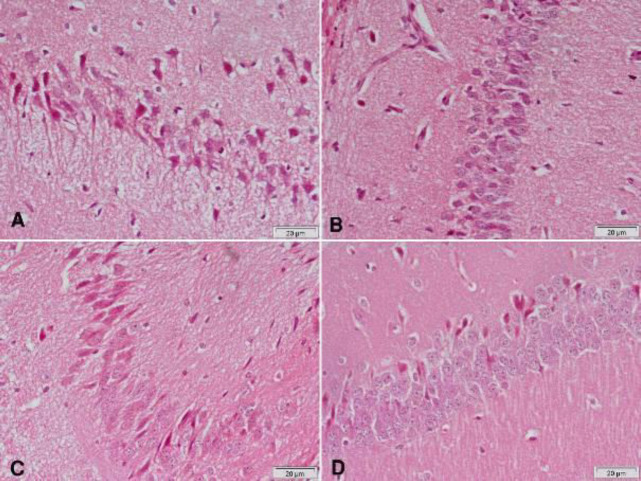
Representative photomicrographs of hippocampal area sections stained with Hx & E after treatment with (A) Rotenone: decrease of the thickness of this area with disorganization of neurons that appear small. Most of them are strongly eosinophilic with pyknotic nuclei. (B) Rotenone+curcumin: marked decrease of damaged neurons. (C) Rotenone+L-dopa: some neurons appear darkly stained with pyknotic nuclei. (D) Rotenone+L-dopa+curcumin: only a few eosinophilic neurons with pyknotic nuclei are seen

**Figure 10:**
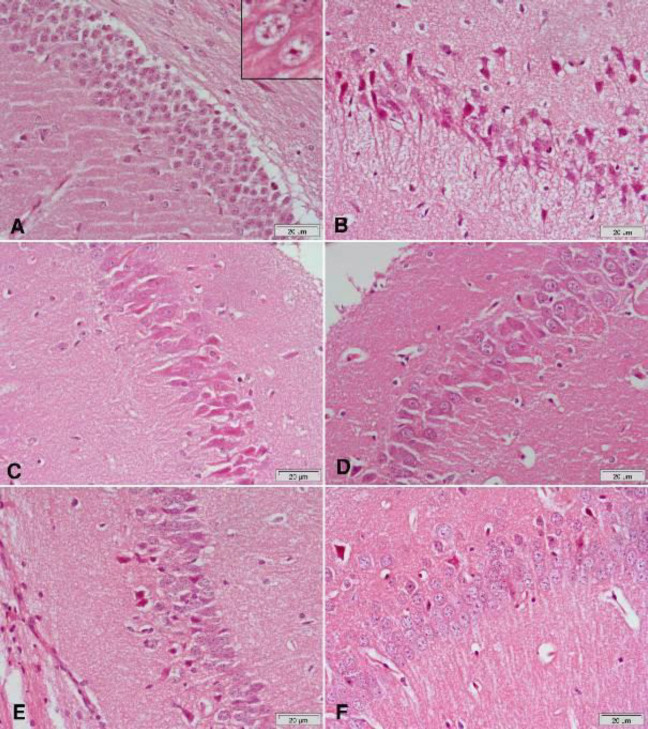
Representative photomicrographs of hippocampal area sections stained with Hx & E

Hematoxylin and eosin-stained sections from the substantia nigra of rotenone-only treated rats showed a decrease in the number and size of pigmented neurons (Figure 5A & 6B) that appeared small and oval (Figure 6C) compared with the vehicle-treated group with large pigmented neurons (Figure 6A). These changes were ameliorated by treatment with curcumin (Figure 5B & 6E), L-dopa (Figure 5C), or rasagiline (Figure 6D) while marked improvement in the number and size of pigmented neurons was observed after treatment with the combination of curcumin/L-dopa (Figure 5D) or curcumin/rasagiline (Figure 6F). Sections of the cerebral cortex from vehicle-treated rats showed normal neuronal appearance (Figure 8A). Signs of neurodegeneration were seen in sections from rotenone-only-treated rats (Figure 7A). Neurons appeared smaller in size than normal, deeply stained with eosinophilic cytoplasm (Figure 8B) and pyknotic or fragmented nuclei and focal infiltration with inflammatory cells (Figure 8C). These pathological alterations were ameliorated by curcumin (Figure 7B) or L-dopa (Figure 7C) and to a lesser extent by rasagiline (Figure 8D). The combination of curcumin/L-dopa (Figure 7D) or curcumin/rasagiline (Figure 8F) resulted in nearly normal cerebral cortex tissue. Neurodegenerative changes were also seen in the hippocampus of rotenone-only-treated rats in the form of decreased thickness of this area with disorganization of neurons (Figure 9A). The latter appeared smaller in size, strongly eosinophilic with pyknotic nuclei (Figure 10B & 10C). Curcumin (Figure 9B & 10D) and to a lesser extent L-dopa (Figure 9C) or rasagiline (Figure 10D) given to rotenone-treated rats ameliorated this damaging effect, while more improvement was seen after the combination of curcumin/L-dopa (Figure 9D) or curcumin/rasagiline (Figure 10F).

***Immunohistochemical results***

**Figure 11:**
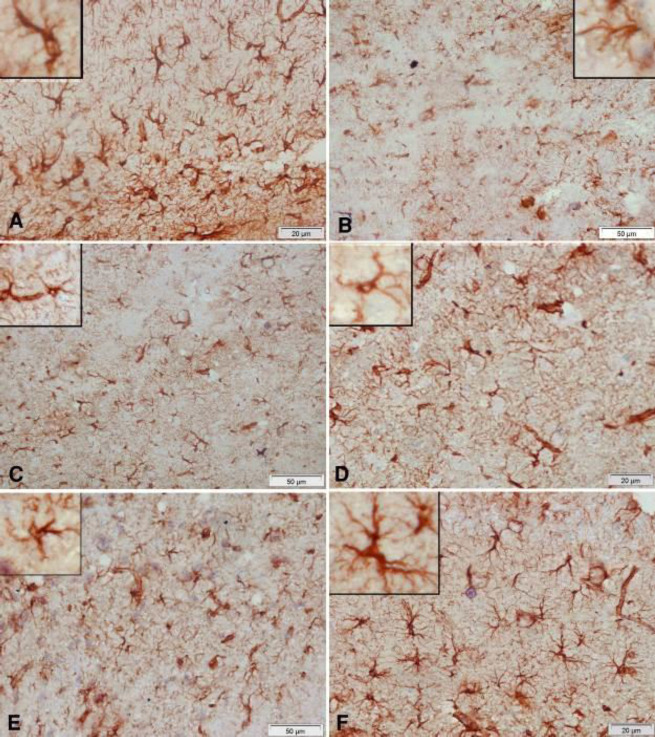
Representative photomicrographs of cerebral cortex sections stained immunohistochemically with GFAP antibody in rats treated with: (A) Vehicle: the positive reaction of glial cells in the normal cerebral cortex. (B) Rotenone: marked decrease in positive reaction. Notice the decrease in cell body size of glial cells. (C) Rotenone+rasagiline: mild increase in positively stained glial cells. (D) Rotenone+L-dopa: mild increase in positively stained glial cells. (E) Rotenone+L-dopa+curcumin: noticeable increase in positively stained cells, though they still have small bodies and short dendrites. (F) Rotenone+rasagiline+curcumin: positive reaction close to normal. Note the large cell body and long dendrites of glial cells in the upper left part of the figure

Compared with the vehicle-treated group, rotenone caused a marked decrease in GFAP reaction in the cerebral cortex. Glial cells appeared smaller and shrunken. Administration of curcumin, L-dopa, or rasagiline resulted in an increase in positively stained glial cells while better amelioration of the rotenone effect was obtained after treatment with the combination of curcumin/L-dopa or curcumin/rasagiline (Figure 11).

## Discussion

Rotenone, a pesticide and a complex I inhibitor is known to reproduce several features of human PD when injected into rodents (13, 24). Our study demonstrates decreased motor activity, coordination, and muscle strength in mice after systemic rotenone administration. Histological investigations revealed a decrease in the size and number of pigmented substantia nigra neurons, neuronal degeneration in the cerebral cortex and hippocampus, and decreased GFAP staining in the cerebral cortex. There was also DNA fragmentation in mice striata. These findings are consistent with previously published studies (25, 26). The results showed that rotenone exceedingly increased MDA and NO levels and decreased GSH in the brain which is an indicator of oxidative stress. Rotenone has been shown to increase the release of reactive oxygen species (ROS) (27) in dopaminergic cell culture *in vitro* (28). It is well known that rotenone acts by interfering with the electron transport chain in mitochondria and increases the generation of mitochondrial ROS such as peroxides, superoxide, hydroxyl radical, and singlet oxygen (24, 29)**. **Rotenone has also been reported to activate microglia cells and these in turn release increased amounts of superoxide and hypochlorous acid (30). ROS can cause many cellular effects such as DNA damage, lipid peroxidation, and protein degradation (31, 28). In addition, mitochondrial dysfunction is associated with increased ROS formation in PD (32). Oxidative stress is thought to have a key role in dopaminergic cell death caused by rotenone. This is because anti-oxidants such as α-tocopherol, ascorbic acid, or the glutathione precursor *N*-acetylcysteine were found to afford protection against rotenone both *in vitro* and *in vivo* (33).

The present study also shows increased brain nitric oxide (NO) levels following rotenone injections. This observation is consistent with other published studies (34, 35). Under physiological conditions, brain NO has an important role as a neurotransmitter and vasodilator. Nitric oxide is synthesized from L-arginine via the action of the enzyme nitric oxide synthase (NOS). While, the release of small amounts of NO by the neuronal (nNOS) and endothelial (eNOS) isoforms mediates the physiological actions of NO, higher amounts generated for a prolonged time by the inducible isoform (iNOS) are neurotoxic (36). This occurs during inflammation where there is increased expression of iNOS in the immune and glial cells. The mechanisms by which NO causes neuronal injury involve oxidative/nitrosative stress through the formation of the highly reactive peroxynitrite or nitrogen oxides, resulting in inhibition of mitochondrial respiration and energy depletion (37). Rotenone was shown to increase the expression of iNOS in the substantia nigra and striatum and to increase brain levels of NO (27, 34, 38). A role for NO in rotenone neurotoxicity is supported by studies indicating a protective effect for nNOS or iNOS inhibitors (39). 

When injected into rodents, rotenone causes motor deficits reminiscent of PD (25, 26). In this study, rotenone suppressed spontaneous vertical exploration or the rearing activity. The latter is affected by the animal’s emotional state and used as a measure of anxiety-like behavior with those who explore less being anxious (40). Rearing is also a marker of environmental novelty where the hippocampal formation is important in controlling rearing behavior in novel environments (41). Hence rearing could be influenced by hippocampal damage which is seen in our histological study. Rearing activity also reflects motor function and hence brain function (42). In addition to decreasing exploratory activity, rotenone resulted in decreased grip strength, skilled reaching, and muscle coordination as indicated by the wire-hanging, stair, and wood-walking tests, respectively. These observations are in agreement with previous studies (25, 26, 43). Rotenone injection in rats induces a hypokinetic behavior that is L-dopa responsive (40). Moreover, Cannon *et al*. (2009) found that the behavioral deficits in rotenone-treated rats such as the decrease in motor activity and spontaneous rearing, and postural instability were responsive to administering a dopaminergic agonist, suggesting that these motor impairments are related to the degeneration of dopaminergic neurons (25). 

Curcumin, the major polyphenolic component from *Curcuma longa *is gaining wide interest as a potential remedy for neurodegenerative disorders because of its anti-oxidant and anti-inflammatory properties (10, 44). We, therefore, investigated the effect of co-administering curcumin in the rotenone model of PD in mice. We found curcumin alleviated the increase in lipid peroxidation and nitric oxide and the decrease in reduced glutathione in the brain of rotenone-treated animals. It also increased the number of spontaneous rears and grip strength and prevented locomotor abnormalities in the stair and wood-walking tests. Studies indicated that curcumin exerts an anti-oxidant effect (45), being more potent than vitamin E in scavenging free radicals (46). It has also nitric oxide scavenging action (47). Curcumin treatment decreased intracellular ROS induced by mutant A53T alpha-synuclein *in vitro*, a PD model (48), and reduced oxidative damage in models of Alzheimer’s disease (49). The neuroprotective effects of curcumin can therefore be ascribed to its anti-oxidant properties (45, 50) whereby, the oxidizing actions of ROS such as superoxide, hydroxyl radical, and others on the cell biomolecules are prevented and which in turn results in less neuronal damage or death. Curcumin has also been shown to exert iron-chelating effects in the 6-hydroxydopamine model of PD (50). An increase in nigrostriatal iron is found in human PD (42) and is considered to contribute to oxidative stress via the Fenton reaction (51, 52). Our histological study supports the neuroprotective effect of curcumin. We have shown that curcumin treatment was shown to maintain the number and size of pigmented dopaminergic substantia nigra neurons. Rotenone produces loss of tyrosine hydroxylase positive neurons and decreases immunohistochemical expression of tyrosine hydroxylase in substantia nigra and striatum (25, 35). Curcumin was able to attenuate the rotenone-induced depletion in striatal tyrosine hydroxylase content. This finding provides further support that treatment with curcumin protected against the loss of dopaminergic neurons after rotenone injection. Rotenone causes damage to other brain regions e.g., the cerebral cortex and hippocampus as shown in this study and previous reports. 

Currently, the pharmacological treatment of PD is based on correcting the deficit in brain dopamine by administering the dopamine precursor L-dopa. Other drugs such as the irreversible MAO-B inhibitors selegiline and rasagiline reduce the metabolism of dopamine and thus increase its concentration and duration of action in the synapse (53). Rasagiline has been shown to exert neuroprotective effects in models of PD, decreasing cell death via anti-apoptosis (54) or increased glial cell line-derived neurotrophic factor (55). In this study, the possible modulatory effect of curcumin on the action of the standard antiparkinsonian drug L-dopa or with the MAO-B inhibitor rasagiline was investigated. We found that administration of either L-dopa or rasagiline to rotenone-treated mice was associated with reduced levels of brain oxidative stress, increased rearing activity, and improved motor coordination in the stair and wood-walking tests. Moreover, treatment with L-dopa increased grip strength. However, the results of the histological study and striatal tyrosine hydroxylase indicate a mild effect for either L-dopa or rasagiline in protecting substantia nigra neurons as compared with that of curcumin. On the other hand, a higher degree of neuroprotection is clearly shown upon co-administering curcumin with either L-dopa or rasagiline compared with either drug alone, indicating an additive effect for curcumin. 

Astrocytes, the most abundant type of glial cells in the brain, express GFAP, a structural protein in glial filaments (56). GFAP is also a marker for glia activation in a number of brain insults (57). In this study, rotenone caused decreased GFAP staining and cell body size of astrocytes in cerebral cortex sections. This finding is consistent with previous studies and suggests astrocytic degeneration or death due to rotenone (26). This effect of rotenone was alleviated upon administering L-dopa or rasagiline, thereby, indicating a neuroprotective effect for these agents. A marked increase in GFAP staining was however evident on co-administering curcumin with either L-dopa or rasagiline. 

DNA ladder assay showed DNA fragmentation in ladder pattern upon mice rotenone treatments. This result is in agreement with the results of both Swarnkar with his co-workers (2012) (58) and Jia *et al*., (2010) (59); where they recorded another type of DNA damage that increased DNA strand breaks upon rotenone treatment in cell lines. However, DNA damage was quenched in the present study by co-treatments by curcumin, rasagiline, and L-DOPA

 in rotenone-treated mice. Additionally, co-treatments by curcumin in combination either with rasagiline or L-DOPA in rotenone-treated mice showed a protective effect on DNA that appeared as a good intact band without fragmentation. 

*: *P<*0.05 vs corresponding vehicle-treated group. +: vs5 vs rotenone control group. #: *P<*0.05 vs rasagiline

*: vs5 vs corresponding vehicle-treated group. +: vs5 vs rotenone control group

*: vs5 vs corresponding vehicle-treated group and between different groups as indicated on the graph. +: vs5 vs rotenone control group. #: vs5 vs rasagiline or rasigline+curcumin (3A) or vs rasagline (3B)

(A) Rotenone: decreased number and size of neurons. (B) Rotenone+curcumin: increase in number and size of characteristic cells. (C) Rotenone+L-dopa: mild increase in the size of neurons. (D) Rotenone+curcumin+L-dopa: noticeable increase in the number and size of neurons

(A) Vehicle: normal appearance of large pigmented neurons. (B) Rotenone: decrease in number and size of neurons. (C) Rotenone: marked decrease of pigmented neurons that appear small and oval. (D) Rotenone+rasagiline: mild increase in the size of neurons. (E) Rotenone+curcumin: marked increase in number and size of characteristic cells. (F) Rotenone+rasagiline+curcumin: quite normal structure of this area

(A) Rotenone: many neurons appear smaller than normal and deeply stained. The upper left part of the figure shows neurons with eosinophilic cytoplasm and pyknotic or fragmented nuclei. (B) Rotenone+curcumin: reduction of damaged neurons if compared with the previous group. (C) Rotenone+L-dopa: noticeable decrease of small dark cells. (D) Rotenone+curcumin+L-dopa: most neurons are normal

(A) Vehicle: normal structure of neurons with characteristic large vesicular nuclei. (B) Rotenone: many neurons appear smaller than normal and deeply stained. The upper left part of the figure shows neurons with eosinophilic cytoplasm and pyknotic or fragmented nuclei. (C) Rotenone: focal infiltration with inflammatory cells. (D) Rotenone and rasagiline: amelioration of the damaging effect of rotenone, though some small dark neurons (arrows) and karyorrhectic (arrowheads) are still observed. (E) Rotenone and curcumin: most of the neurons appear normal with few small dark neurons. (F) Rotenone+rasagiline+curcumin: quite normal cerebral cortex tissue

(A) Vehicle: normal structure of this area. (B & C) Rotenone: decrease in thickness of this area with disorganization of neurons that appear small. Most of them are strongly eosinophilic with pyknotic nuclei. (D) Rotenone+rasagiline: no pyknotic nuclei are seen, although many neurons still show darkly-stained cytoplasm. (E) Rotenone+curcumin: marked decrease of small dark neurons. (F) Rotenone+rasagiline+curcumin: only a few eosinophilic neurons with pyknotic nuclei

## Conclusion

This study indicates that curcumin could be absorbed and reached the brain at concentrations sufficient to exert biochemical changes and prevent DNA fragmentation caused by rotenone in mice. Curcumin improved motor power and coordination, alleviated the death in substantia nigra neurons, and maintained striatal tyrosine hydroxylase content. Curcumin showed some additive effects to L-dopa or rasagiline. Curcumin thus might be useful as an adjuvant treatment for PD. We recommended the use of curcumin as an anti-oxidant medication especially in geriatric persons due to its high neuroprotective effect. 

## Authors’ Contributions

OMEAS Conceived the study, design and performed data analysis; MESES Prepared drugs and performed experiments, behavioral tests, biochemical assays, and data analysis; HMZ Assessed DNA fragmentation and provided interpretation; NS Performed histopathological studies and their interpretation; OMEAS, MESES, HMZ, and NS Helped with manuscript preparation, revision, and final approval of the version to be published.

## Conflicts of Interest

The contributed authors have no potential conflicts of interest.
